# Genetic Diversity Analysis of Coxsackievirus A8 Circulating in China and Worldwide Reveals a Highly Divergent Genotype

**DOI:** 10.3390/v12101061

**Published:** 2020-09-23

**Authors:** Yang Song, Dongyan Wang, Yong Zhang, Zhenzhi Han, Jinbo Xiao, Huanhuan Lu, Dongmei Yan, Tianjiao Ji, Qian Yang, Shuangli Zhu, Wenbo Xu

**Affiliations:** 1 WHO WPRO Regional Polio Reference Laboratory, National Health Commission Key Laboratory for Medical Virology, National Institute for Viral Disease Control and Prevention, Chinese Center for Disease Control and Prevention, No. 155, Changbai Road, Changping District, Beijing 102206, China; candyalbarn57@126.com (Y.S.); yanzi1973_55@163.com (D.W.); hansir8@sina.com (Z.H.); mr_mint1114@sina.com (J.X.); luhuanhuan0908@163.com (H.L.); dongmeiyan1976@163.com (D.Y.); jtj112@163.com (T.J.); yangqian@ivdc.chinacdc.cn (Q.Y.); zhusli@126.com (S.Z.); 2 Center for Biosafety Mega-Science, Chinese Academy of Sciences, Wuhan 430071, China

**Keywords:** coxsackievirus A8, emerging diseases, evolutionary dynamics, genetic diversity, hand, foot, and mouth disease, phylogenetic analysis

## Abstract

Coxsackievirus A8 (CV-A8) is one of the pathogens associated with hand, foot and mouth disease (HFMD) and herpangina (HA), occasionally leading to severe neurological disorders such as acute flaccid paralysis (AFP). Only one study aimed at CV-A8 has been published to date, and only 12 whole-genome sequences are publicly available. In this study, complete genome sequences from 11 CV-A8 strains isolated from HFMD patients in extensive regions from China between 2013 and 2018 were determined, and all sequences from GenBank were retrieved. A phylogenetic analysis based on a total of 34 complete *VP1* sequences of CV-A8 revealed five genotypes: A, B, C, D and E. The newly emerging genotype E presented a highly phylogenetic divergence compared with the other genotypes and was composed of the majority of the strains sequenced in this study. Markov chain Monte Carlo (MCMC) analysis revealed that genotype E has been evolving for nearly a century and somehow arose in approximately 2010. The Bayesian skyline plot showed that the population size of CV-A8 has experienced three dynamic fluctuations since 2001. Amino acid residues of *VP1*_100N_, _103Y_, _240T_ and _241V_, which were embedded in the potential capsid loops of genotype E, might enhance genotype E adaption to the human hosts. The CV-A8 whole genomes displayed significant intra-genotypic genetic diversity in the non-capsid region, and a total of six recombinant lineages were detected. The Chinese viruses from genotype E might have emerged recently from recombining with European CV-A6 strains. CV-A8 is a less important HFMD pathogen, and the capsid gene diversity and non-capsid recombination variety observed in CV-A8 strains indicated that the constant generation of deleterious genomes and a constant selection pressure against these deleterious mutations is still ongoing within CV-A8 quasispecies. It is possible that CV-A8 could become an important pathogen in the HFMD spectrum in the future. Further surveillance of CV-A8 is greatly needed.

## 1. Introduction

Human enteroviruses (EVs) are genetically diverse RNA viruses belonging to the genus *Enterovirus* and family *Picornaviridae*. There are currently over 100 designated human EVs that are classified into four species: EV-A to -D. The EV-A species currently consists of 25 serotypes, including coxsackievirus A8 (CV-A8) [1]. Similar to all EVs, CV-A8 is a small, non-enveloped, single stranded, positive-sense RNA virus. The genome, which has approximately 7400 nucleotides (nt), contains a long open reading frame (ORF) flanked by a 5′-untranslated region (*UTR*) and a 3′-*UTR*. The ORF can be translated into a 2,189-amino acid-long polyprotein and then cleaved into the three polyprotein precursors *P1*, *P2*, and *P3*, which encode the structural proteins *VP4*, *VP2*, *VP3*, and *VP1*, and the nonstructural proteins *2A*, *2B*, and *2C*, and *3A*, *3B*, *3C*, and *3D* [2]. Phylogenetic trees based on the complete *VP1* capsid sequences of EVs have been used for discrimination of genotypes, as the *VP1* region is surface exposed, contains many important neutralisation epitopes and is serotype specific. Based on this approach, several EV serotypes, such as EV-A71, CV-A16, CV-A6, CV-A4, and CV-A2, have been genotyped [3,4,5,6,7,8].

CV-A8 infections are associated with a wide spectrum of illnesses, including mild diseases such as febrile illness, hand, foot, and mouth disease (HFMD) and herpangina (HA), but occasionally lead to severe neurological disorders such as acute flaccid paralysis (AFP) [9,10,11]. The CV-A8 prototype Donovan strain was first isolated from the United States in 1949 [1]. CV-A8 has been reported to be associated with sporadic AFP cases in China and India [10,12], respiratory cases in Kenya [13], and some clinical cases in The Netherlands [14]. An atypical paediatric case of lamellar ichthyosis was reported by CV-A8 infection [15]. Additionally, sewage surveillance in the Philippines and western India documented the detection of CV-A8 [9,16]. In Thailand, CV-A8 was the most prevalent cause of HA in 2012 [17]. In China, CV-A8-related HFMD and HA cases have been reported in many provinces. It has been reported that in Guangdong, China, the usual CV-A8 strains were detected during hospital-based surveillance for HFMD from 2012 to 2014, and CV-A8 was strongly associated with several HA outbreaks in nursery schools in 2013 [11,18]. These clinical findings highlighted that CV-A8 infections might be associated with multiple types of diseases, posing a disease burden to global public health, especially for young children. Therefore, a greater understanding of this pathogen is important.

There has been only one study describing the genomic characteristics of CV-A8 to date [11], and only 12 whole-genome sequences are publicly available. Two published studies have characterized the *VP1* phylogeny of CV-A8 but were based only on short partial *VP1* sequences [11,17]. Both studies might have limitations and did not reach the same result but can be well referenced for our research on CV-A8 genotyping. In this study, we contribute the full-length genome sequences of 11 CV-A8 isolates from paediatric HFMD patients from six provinces and municipalities of China detected between 2013 and 2018 and retrieve all the CV-A8 sequences from the GenBank database to characterize the genetic diversity, evolutionary dynamics and recombination patterns of CV-A8.

## 2. Materials and Methods

### 2.1. Virus Isolation

The clinical HFMD samples were collected from the HFMD Surveillance Network established in our laboratory from 2008 to 2018. The samples were processed based on standard protocols [19] and were first confirmed as positive for EV by a commercial real-time PCR assay (Shuoshi Biotech, Taizhou, Jiangsu, China). All the above EV-positive samples were then inoculated into human rhabdomyosarcoma (RD) and human laryngeal epidermoid carcinoma (HEp-2) cell lines for virus propagation and purification. Infected cell cultures were harvested after a complete cytopathic effect (CPE) was observed.

### 2.2. CV-A8 Whole-Genome Sequencing

Viral RNA was extracted using a QIAamp Viral RNA Mini Kit (Qiagen, Valencia, CA, USA). First, we performed reverse transcription polymerase chain reaction (RT-PCR) to amplify the *VP1* capsid region using a PrimeScript One Step RT-PCR Kit Ver. 2 (TaKaRa, Dalian, China) with all-purpose EV-A primers designed in our laboratory. The PCR products were purified using a QIAquick PCR Purification Kit (Qiagen, Hilden, Germany), and then amplicons were bidirectionally sequenced using an ABI 3130 Genetic Analyzer (Applied Biosystems, Foster City, CA, USA). Second, a neighbour-joining tree of *VP1* combined with the EV prototype *VP1* sequences was constructed using MEGA (v7.0) for determining CV-A8 samples [20]. Eleven samples isolated between 2013 and 2018 were identified as CV-A8; no strains isolated before 2013 were identified as CV-A8.

The 5′ end of the genome sequence was amplified using a 5′-Full RACE Kit (Takara Biomedicals, Dalian, China), and the 3′-end sequence was obtained using an oligo-dT primer (primer 7500A) [21] as the downstream primer for amplification. The primers used for PCR amplification and sequencing of the remaining genome in this study were designed based on the primer walking method (Supplementary Table S1).

### 2.3. Dataset Construction

In addition to the 11 samples sequenced in this study, all CV-A8 *VP1* and (near) whole-genome sequences (dated to 1 June 2020) in the GenBank database were retrieved, including 23 complete *VP1* sequences, 160 partial *VP1* sequences that shared the same region of 155 bp, and 12 whole-genome sequences. A total of 34 complete *VP1*, 171 partial *VP1* and 23 whole-genome sequences constituted the CV-A8 dataset.

### 2.4. Phylogenetic and Evolutionary Analyses of CV-A8 VP1 Sequences

Sequence alignment was conducted using the Muscle tool in MEGA (v7.0). RAxML (v8.2.12) was used to construct maximum likelihood trees for 34 entire and 171 partial CV-A8 *VP1* sequences [22]. The CV-A3 prototype strain was used as the outgroup for constructing the CV-A8 ML tree for genotyping. The best nucleotide substitution models “GTR+G” and “K2+G” were selected by jModelTest (v 2.1.7) for each dataset [23]. Support was estimated with 1000 bootstrap replicates, and the results were visualized using FigTree (v1.4.4). The methodology of genotyping was based on enterovirus 71 (EV-A71) genotypes, which were defined using a 15–25% divergence threshold for the *VP1* coding region. In addition, a neighbor-joining tree, a minimum evolution tree and an UPGMA tree were also constructed using the Kimura 2-parameter model with 1000 bootstrap replicates for 34 CV-A8 entire *VP1* sequences and the CV-A3 prototype strain. The Markov chain Monte Carlo (MCMC) method implemented in BEAST (v1.10, Los Angeles, CA, USA) was used to estimate the temporal phylogenies and rates of evolution, the ML tree was imported as the specified starting tree, and the operations of fixed tree topology were selected when auto optimizing [24]. The 34 *VP1*-region sequences were analysed using the uncorrected lognormal clock (UCLD) and constant site tree prior to the GTR+G nucleotide substitution model. A Bayesian MCMC run of 1 × 10^8^ generations was implemented with a sampling frequency of 1 × 10^4^ generations. The output from BEAST was analysed using TRACER (v1.7.1). A maximum clade credibility (MCC) tree was constructed using TreeAnnotator, with the burn-in option used to remove the first 10% of sampled trees. A Bayesian skyline plot was inferred using the same clock and nucleotide substitution model to reconstruct the evolutionary history of CV-A8. A geographic map of China from Highcharts (grant number: 0321912045738052) was used to display the geographic distribution of genotypes of Chinese CV-A8 strains. The protein structure homology-modelling server “SWISS-MODEL” (https://swissmodel.expasy.org/) was used to find the published structural template for the CV-A8 *VP1* capsid [25].

### 2.5. Whole-Genome and Recombination Analyses

The average pairwise genetic diversity along all the CV-A8 genomes was calculated using DnaSP 6 [26] software with a sliding window of 200 nt and a step size of 20 nt. SimPlot (v3.5.1) was used to produce similarity plots with a 200-nt window moving in 20-nt steps to evaluate genetic diversity and detect recombination breakpoints [27]. Recombination was detected using seven algorithms (RDP, Geneconv, BootScan, MaxChi, Chimaera, SiScan, and 3Seq) implemented in RDP4 [28]. Other EV-A strains that had high sequence homology with CV-A8 recombinants in the non-capsid region were screened from GenBank for potential parental strain detection.

### 2.6. Nucleotide Sequence Accession Numbers

All eleven whole-genome sequences of CV-A8 were deposited in the GenBank database under the accession numbers MT648778–MT648788.

### 2.7. Ethics Statement

This study was approved by the Ethics Review Committee (IVDC2016-004, February 2016) of the National Institute for Viral Disease Control and Prevention (IVDC), Chinese Center for Disease Control and Prevention.

## 3. Results

### 3.1. Operational Mechanism of HFMD Surveillance Network and CV-A8 Dataset Overview

As millions of HFMD-related cases and repeated HFMD outbreaks across China were reported in the past decade, an extensive three-level HFMD surveillance laboratory network including one national lab (our lab), 31 provincial labs and 331 prefectural labs was established in China since 2008. Every prefecture laboratory should collect at least five specimens in a month according to standard protocols. Positive samples were sent to provincial laboratories for virus isolation and for the identification of EV-A71, CV-A16 and other EVs (non-EV-A71 and non-CV-A16 EVs). At least 10 EV strains must be isolated in each provincial laboratory monthly; these strains were then sent to our lab for further virus reisolatioin, identification of all the specific EV serotypes and storage.

Eleven CV-A8 strains isolated since 2008 from China were contributed by this study. All the strains were detected from 2013 to 2018, including three from Gansu Province, two each from Jiangxi Province and Tianjin Municipality and one each from Henan, Shandong and Shaanxi provinces and Chongqing Municipality (Table 1). Full-length genome sequences of the 11 samples were acquired. Furthermore, a total of 12 (near) full-length genomes were publicly available from GenBank to date, including the prototype strain from the United States, 1949; eight and one from Guangdong and Zhejiang provinces of China isolated between 2012 and 2014, respectively; and two from Australia, 2017 (Table 1).

**Figure 1:**
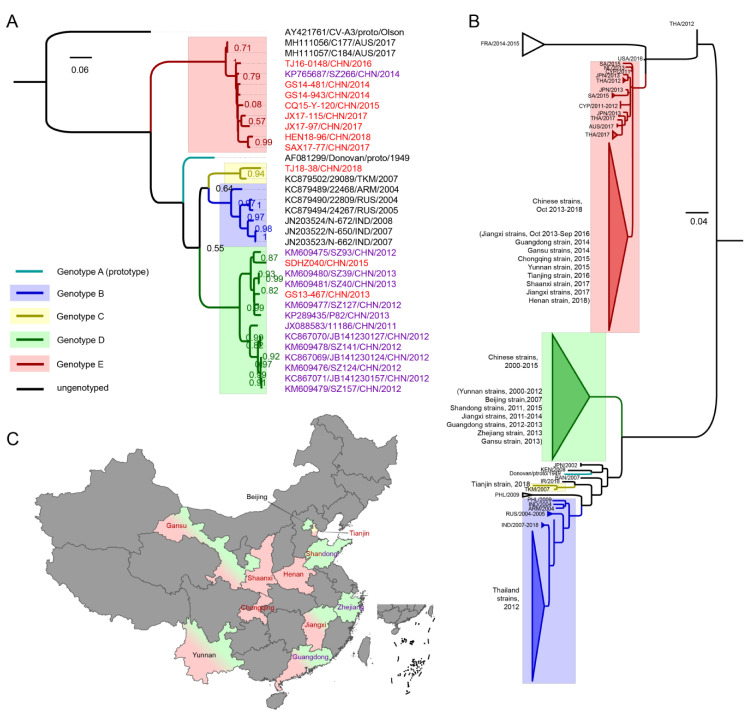
(**A**) Genotyping result of the maximum likelihood phylogenetic tree of CV-A8 strains constructed based on 34 complete *VP1* sequences; the coxsackievirus A3 (CV-A3) prototype strain (AY421761) was used as an outgroup, support was estimated with 1000 bootstrap replicates and the bootstrap cut-off threshold was set to 0.50. The genotypes are differentiated by distinct colours, which are indicated on the left, and the names of the sequences are noted on the right. The Chinese isolates from this study are marked in red, and the Chinese isolates from GenBank are marked in purple. (**B**) Unrooted maximum likelihood phylogenetic tree of 171 global CV-A8 partial *VP1* sequences. Information on the strains detected within each genotype is indicated on the left of the tree. (**C**) The geographical distribution of CV-A8 genotypes in China. The provinces are indicated by the colour(s) of the circulating genotype(s), the name of each province is located on the map, the provinces that provided the complete *VP1* sequence(s) from this study are marked in red and those from GenBank in purple, and the geographic map of China was taken from Highcharts (grant number: 0321912045738052).

All *VP1* sequences (*n* = 34, 11 from this study, 23 from GenBank) were collected and used for CV-A8 genotyping (Table 1). Almost all the partial *VP1* sequences (*n* = 160 from GenBank, 155 bp, dated to 1 June 2020) were retrieved and trimmed for phylogenetic analysis. Notably, among a total of 13 Chinese entirely *VP1* sequences from GenBank, eleven (85%) were from Guangdong Province, whereas the 11 sequences contributed by this study were from seven different provinces and municipalities; hence, this study improved the geographical representation of Chinese CV-A8 sequences used for genetic analyses (Figure 1C).

### 3.2. VP1 Phylogenetic Analysis and Genotyping of CV-A8

A maximum likelihood (ML) tree based on the complete *VP1* sequence of CV-A8 was generated; the CV-A3 prototype strain was used as an outgroup (Figure 1A). The phylogeny revealed five highly bootstrap-supported genotypes: A (*n* = 1), B (*n* = 6), C (*n* = 2), D (*n* = 14) and E (*n* = 11) with high group mean distances varying from 16.3% (genotype B to C) to 24.5% (genotype D to E), indicative of evolutionary distances. Genotype A was formed solely by the prototype Donovan strain isolated in 1949; genotype B consisted of isolates from India, Russia and Armenia detected from 2004 to 2008; genotype C contained a Turkmenistan strain isolated in 2007 and a Chinese strain isolated in 2018; genotype D was composed only of Chinese strains isolated from 2011 to 2015; and genotype E comprised the latest strains from China and Australia isolated from 2014 to 2018. It is interesting that the emerging genotype E formed a highly divergent sister clade for the other genotypes, as shown by the ML tree. When using the neighbor-joining, minimum evolution and an UPGMA methods, the phylogenetic trees all formed almost the same topologies as the ML tree (Supplementary Figure S1). This finding is rare among enteroviruses, as the prototype strain is always the most divergent strain among the genotyping results [3,4,5,6,7,8]. The 24 Chinese strains fell into the three genotypes, C, D and E, suggesting that the circulation of CV-A8 in China had distinctive evolutionary routes. The Tianjin strain isolated in 2018 from genotype C was probably imported because genotype C strains were rare, and only this strain belonged to genotype C. Intriguingly, among the Chinese strains, 12 out of 13 GenBank strains belonged to genotype D, with the exception of one Guangdong strain isolated in 2014 belonging to E, whereas 8 out of 11 strains from this study composed the majority of genotype E strains. This finding indicated that in China, the CV-A8 strains from the highly divergent genotype E have been emerging and that the predominant genotype has undergone a switch from D to E in recent years. Aside from the Chinese strains, two Australian strains isolated in 2017 also clustered with genotype E, suggesting that the circulation of genotype E might be on a global scale.

The phylogenetic tree of 171 partial *VP1* genes was further analysed (Figure 1B, Supplementary Figure S2 and Supplementary Table S2). Although the sequences were too short to present a robust topology, an outline of the three major genotypes—B, D and E—could be summarized. We found that a large number of Thailand strains associated with HA cases in 2012 clustered with genotype B and descended from the Russian and Indian strains; however, no strains detected after 2012 and detected in China belonged to genotype B. Genotype D was found only in China and probably originated from the CV-A8-related AFP cases in Yunnan Province since 2000. Surprisingly, genotype E comprised most of the emerging worldwide strains since 2011, including a large number of Chinese strains detected from late 2013 to 2018. There were three strains from genotype E detected in 2011, including two Cyprus strains and one Netherland strain; a few Thailand strains of HA cases detected in 2012 also belonged to genotype E. The information of the partial *VP1* tree reflected that 21 samples were isolated between 2000 and 2010, but none of them belonged to genotype E; whereas most of the 149 samples detected after 2010 belonged to genotype E (Supplementary Table S2). Furthermore, according to the information of published partial *VP1* sequences, the years of virus isolation have been almost continuous since 2000, but no genotype E strains were detected before 2011 worldwide and 2013 in China; the gradual switch of the genotypes was apparent.. The partial *VP1* phylogeny indicated that the dominant genotype of CV-A8 outside China has been changing from genotype B to E and inside China from genotype D to E in recent years; in other words, the predominant genotype(s) of CV-A8 worldwide has been undergoing a switch from the co-circulation of genotypes B and D to the circulation of genotype E.

### 3.3. The Evolutionary Dynamics of CV-A8 Genotypes

**Figure 2:**
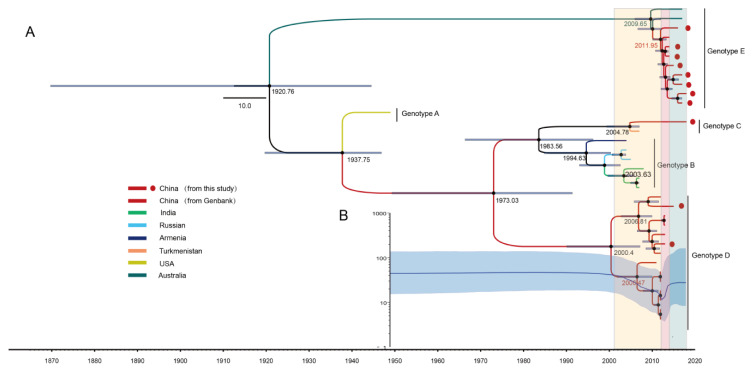
(**A**) The maximum clade credibility (MCC) phylogenetic tree generated using the Markov chain Monte Carlo (MCMC) method based on the complete *VP1* sequences of 34 CV-A8 variants and coloured according to different countries. The scale bar represents time in years. The tree was node-labelled with inferred dates of lineage splits. Each genotype is noted on the right. (**B**) Bayesian skyline plot of the 34 CV-A8 *VP1* region sequences, reflecting the relative genetic diversity from 1949 to 2018. The *x*-axis is the time scale (years), and the *y*-axis is the effective population size. The solid line indicates the median estimates, and blue shading indicates the 95% highest posterior density. The three stages of effective population size are shaded with light yellow, rose and aqua.

The MCC trees based on the 34 CV-A8 complete *VP1* sequences were generated using the MCMC method (Figure 2A). The computed MCC tree revealed that genotype E has been evolving for nearly a century and then somehow emerged in approximately 2010. The evolutionary substitution rate for the *VP1* region of CV-A8 was 4.48 × 10^−3^ (95% highest posterior density (HPD) (2.80–6.14)) × 10^−3^ substitutions site^−1^ year^−1^, with a predicted date of tMRCA at 1921 (95% HPD 1870–1945). Genotype A was suggested to have diverged in approximately 1938, and the tMRCAs of genotypes B, C and D could be traced back to 1995, 2005 and 2000, respectively.

Furthermore, a Bayesian skyline plot analysis was performed to reconstruct the demographic history of CV-A8 based on the 34 complete *VP1* sequences (Figure 2B). The effective population size of CV-A8 strains was constant until 2001, mainly because of the lack of sequence data; however, with the increasing number of CV-A8 strains detected after 2001, the effective population size exhibited three stages. In the first stage of the co-circulation period of genotypes B and D from 2001 to 2012, the population size presented a decrease, possibly due to a reduction in the prevalence of genotype B (or potentially a lack of *VP1* sequences from genotype B). In approximately 2012, the population size underwent rapid growth within 2 years until 2014, corresponding to the second stage. During this time, not only was genotype D predominant but also many strains from genotype E had started to be detected, corresponding to a gradual switch of the predominant genotype of CV-A8. Starting in 2014, with a stable increase in the effective population size, the third stage suggested that genotype E had been dominating the circulation, while genotype D had been decreasing but was still detectable. The genotype switch is still ongoing, which corresponds to the reported surveillance data.

### 3.4. Amino Acid Characterization of the CV-A8 VP1 Capsid

The overall mean amino acid (aa) distance among the 34 CV-A8 *VP1* genes was 4.2%, and the intra-genotypic mean distances of genotypes B, C, D and E were 2.1%, 0.3%, 1.6% and 1.4%, respectively. Between each genotype, the mean distance ranged from 2.3% (genotype A to B) to 6.4% (genotype D to E). The relatively low aa difference compared with the relatively high nucleotide distance indicated that most of the *VP1* capsid nucleotide mutations were synonymous. A total of 43 polymorphic sites were detected among all strains, of which nine sites were specific to genotype E strains (at least nine strains from genotype E had this aa site) (Table 2). In addition, we positioned the potential exposed loops for the CV-A8 *VP1* capsid according to its most similar structure, the CV-A-10 capsid [25,29] (Table 2). Notably, the specific genotype E sites 100N and 103Y embedded in the assumed surface-exposed BC loop and 240T and 241V embedded in the assumed surface-exposed HI loop might enhance genotype E adaption to the human hosts and play an important role in the transmissibility of recently emerging genotype E strains.

### 3.5. Whole-Genomic Diversity of CV-A8

All 23 whole-genome sequences of CV-A8 were analysed, including the prototype strain, one Chinese strain from genotype C, eleven Chinese strains from genotype D, and eight Chinese strains and two Australia strains from genotype E. Except for the Australian strains that lacked partial *UTR* sequences, the full-length genomes were composed of 7394 to 7400 nt, and the varying lengths were led by the variegated *UTR* nucleotides of each sequence.

**Figure 3:**
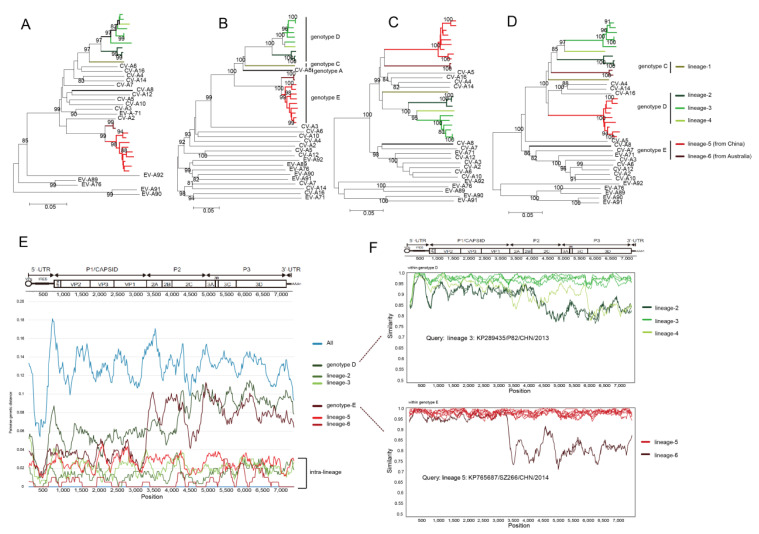
Neighbour-joining phylogenetic trees of 23 CV-A8 variants combined with EV-A prototype strains constructed based on the (**A**) 5′-untranslated region, (**B**) *P1* capsid region, (**C**) *P2* non-capsid region and (**D**) *P3* non-capsid region. The recombinant lineages are differentiated by distinct colours, and each genotype is indicated on the right side of the *P1* tree. (**E**) Average pairwise diversity based on 23 whole genomes of all genotypes, genotypes D and E each, and the intra-genotype recombinant lineage each, using a sliding window of 200 nt with a step of 20 nt. (**F**) Recombination breakpoints based on the whole genomes of different lineages as detected within genotypes (**D**,**E**).

NJ trees were constructed based on the *5′UTR*, *P1* capsid, *P2* and *P3* non-capsid region of the 23 CV-A8 variants combined with the other EV-A prototype strains (Figure 3A–D). Obvious discrepancies were reflected, suggestive of polyphyletic recombination among CV-A8 variants. However, the recombination within each genotype was not monophyletic either. Taking the non-capsid *P3* phylogeny as an example, within genotype D, the tree indicated three independent lineages (Figure 3D), implying that three diverse recombination events occurred among genotype D strains; genotype E even reflected more apparent phylogenetic violation in that the Chinese strains and the Australian strains were separated into two lineages by other EV-A prototype strains, which suggested distinct evolutionary routes. The strains that formed each recombinant lineage in the *P3* region also clustered the most closely together in the *5′UTR* and *P2* region, which indicated intra-lineage whole-genomic monophyly. Therefore, genotypes D and E contained three and two recombinant lineages, respectively, which were designated as recombinant lineages 2–6. The Tianjin strain from genotype C was named lineage 1 (Figure 3A–D).

We further employed a sliding window analysis using the DnaSP package of CV-A8 whole genomes (Figure 3E) and found that the pairwise genetic diversity was high throughout the whole genomes when calculating all the genotypes together. For genotypes D and E, the pairwise genetic diversity in the *P2* and *P3* regions was obviously higher than in the *P1* region, suggesting a monophyletic capsid region but a polyphyletic non-capsid region. However, when computing within the intra-genotype lineage (except for lineage 4, which contained only one strain), the pairwise genetic diversity was low from beginning to end. The recombination breakpoint of each recombinant lineage was further positioned within the genotype using similarity scanning (Figure 3F), which indicated obvious breakpoints between/among intra-genotypic recombination lineages.

### 3.6. Multiple Recombination Events Were Detected among CV-A8 Variants

**Figure 4:**
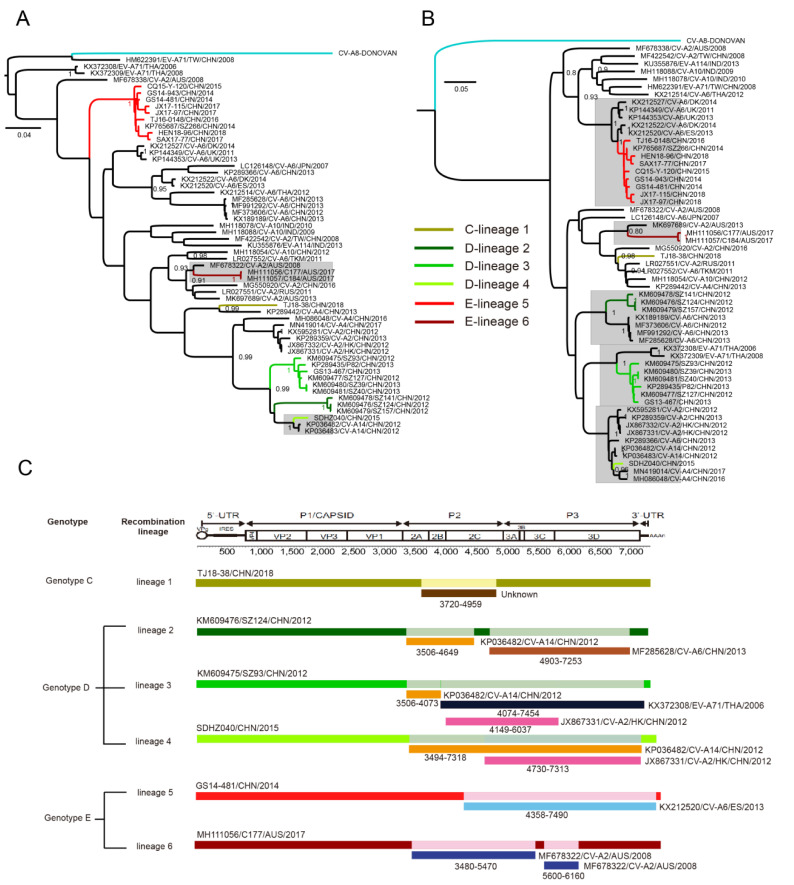
Maximum likelihood phylogenetic trees of 23 CV-A8 variants combined with 35 other EV-A strains screened from GenBank constructed based on the (**A**) *P2* non-capsid region and (**B**) *P3* non-capsid region. The CV-A8 recombinants and some of the other serotype strains which clustered closely together and formed a clade with a high bootstrapped value were shaded with grey background, the prototype strain was marked in blue. (**C**) Specific recombination event detection using the RDP4 software package.

To find the potential parental recombination strains for each lineage, the other 35 non-CV-A8 EV-A strains that had high sequence homology with each lineage in the non-capsid region were screened from GenBank for detection. We used the *2A*, *2B*, *2C*, *3AB*, *3C* and *3D* non-capsid region sequences of each lineage, respectively, for BLASTing on the Genbank database, screening the other non-CV-A8 EV-A serotype strains that had high sequence homology (at least more than 88%) with each intra-lineage non-capsid sequence of CV-A8. The specific recombination events were analysed using two strategies: a manual approach using phylogenetics and genetic distances and a more automatic approach using the RDP4 software package. Notably, from the ML trees generated based on *P2* and *P3*, the *P3* region sequences in particular, the CV-A8 recombinants and some of the other serotype strains clustered closely together and formed a clade with a high bootstrapped value (Figure 4A,B), indicating the possibility of various inter-serotypic recombination events in the non-capsid region.

Notably, the computed result of RDP4 (Figure 4C) indicated the newly emerging Chinese strains of lineage 5, genotype E, presented strong recombination evidence with some CV-A6 strains isolated from European countries in the whole *P3* region, with great nucleotide similarity at approximately 95%; however, these CV-A6 recombinants were only detected in some European countries but not in China, and it is likely that these CV-A8 recombinants might have been imported from European countries as many genotype E strains were also detected in European countries based on the partial *VP1* phylogeny. Furthermore, even though the Australian strains of lineage 6 were isolated later than most of the Chinese strains of lineage 5, the MCC tree (Figure 2) reflected that the Australian strains arose earlier than the Chinese strains, which further suggested that Chinese recombinants might be recently generated by recombining with other non-CV-A8 EVs’ non-capsid genes.

## 4. Discussion

In this study, we segregated CV-A8 into five genotypes, namely, A, B, C, D and E, and described the whole-genomic diversity of CV-A8. Among the HFMD samples collected since 2008 from our lab, eleven samples isolated between 2013 and 2018 were identified as CV-A8, and among these 11 samples, eight of them were identified as genotype E strains. From 2008 to 2012, no CV-A8 strains were identified in our lab, maybe because of their lower transmissibility; from 2013 onwards, 11 CV-A8 samples had been identified, probably because the transmissibility of CV-A8 started to increase around that time. Furthermore, 8 of the 11 strains belonged to genotype E. We were aware that, with so many labs doing HFMD-related EVs detection in China, different laboratory capabilities, transporting distances, commercial kits, etc. would lead to inhomogeneity of the strains received by us; nevertheless, such a result would still somehow imply that the genotype E strains from China were newly arisen and might have higher transmissibility. It is intriguing that the newly emerging genotype E strains presented highly divergent *VP1* capsid sequences among the CV-A8 variants; the nine specific aa residues of genotype E, especially 100N, 103Y, 240T and 241V embedded in the assumed surface-exposed loops, might enhance genotype E adaption to the human hosts as surface exposed loops’ structures in the *VP1* capsid always serve as potential viral-neutralizing epitopes for EVs. Further experiments are needed to verify this conjecture. Furthermore, recombination is a frequently observed phenomenon among EVs and, more importantly, has long been recognized to act as a driving force of EV evolution by eradicating deleterious mutations. Because the *3D^pol^* error-prone RNA-dependent RNA polymerases (*RdRps*) of EV always lead to misincorporations during genome replication, ongoing recombination may be the main process preventing EV genomes from deleterious mutation accumulation. In addition, recombination creates chimeric molecules from parental genomes with different phylogenetic origins and may also help EVs attain combined advantageous features from various genomes during the process of evolution [30,31,32]. This may generate new recombinants with higher virulence and transmissibility, such as vaccine-derived polioviruses and the C4a evolutionary branch of EV-A71 [33,34,35,36]. In this study, we found that all the Chinese strains from genotype E, i.e., lineage 5, were likely to emerge recently from recombining with European CV-A6 strains in the whole *P3* region. It is interesting that CV-A6 recombinants were reportedly associated with more severe HFMD clinical manifestations of eczema herpeticum than typical HFMD cases [37], which might be influenced by the non-capsid features conjectured by scholars. Some advantageous non-capsid features might be obtained by the CV-A8 strains of recombinant lineage 5 and somehow impart higher transmissibility. Another interesting phenomenon was observed within Chinese CV-A8 strains of lineage 2, genotype D; this lineage was found to be recombined with two types of Chinese CV-A6 recombinants [38]. These two types of recombinants were associated with clinical features of more widespread skin lesions and severe HFMD [38,39], and the non-capsid features of lineage 2 CV-A8 may affect the pathogenicity of these CV-A6 recombinants. Although CV-A8 strains have composed the minority of HFMD pathogens, they may serve as an important recombination interchange within the EV-A gene pool and help themselves or other EVs attain advantageous features.

Overall, based on the above results, potential reasons for the emergence of genotype E could be that: (i) during the evolution of genotype E, critical aa residues on the capsid region, especially those on the potential exposed loops, underwent mutations to change the capsid structures, making it easier to interact with the receptors and adapt to the host; (ii) recombination in the non-capsid genes might play an important role in the transmissibility of genotype E, in particular in the newly emerging recombinants of lineage 5 detected in China; (iii) due to CV-A8 being a minor component of the HFMD and HA pathogen spectra, few studies have focused on the detection and sequencing of CV-A8 over these years, leading to public resource limitations; (iv) the circulation of CV-A8 strains might have regional differences, especially in China, as most of the complete *VP1* Chinese sequences from GenBank were from southern areas (Guangdong and Zhejiang provinces), whereas the sequences provided by this study were from distributed regions. Even though the first two conclusions were drawn from the results of genetic analyses, the data limitations could not be neglected, as we mentioned in reasons “iii” and “iv”. Therefore, future surveillance and further sequencing of CV-A8 is important for more accurate analyses.

Mainland China incorporated HFMD into the National Notifiable Disease Surveillance System (NNDSS) in 2008, and HFMD has had the highest yearly incidence among all national notifiable diseases since 2010, with over 1.5 million cases annually reported [40,41]. Although EV-A71, CV-A16 and CV-A6 have been commonly regarded as the leading pathogens of HFMD worldwide, other EVs have been frequently reported in recent years, including CV-A8 [41]. CV-A8 was also reported to cause HA, but HA was not enrolled in the disease surveillance reporting system of China; in addition, multiple clinical phenotypes, including AFP, lamellar ichthyosis, respiratory disease, and other features, have been reported to be associated with CV-A8 infection; therefore, the burden of CV-A8-related diseases might have been underestimated, posing a threat to public health. CV-A8 is a less important HFMD pathogen, and such capsid diversity (multiple genotypes) and frequent recombination (different intra-genotypic recombinant lineages) observed in CV-A8 indicate that the CV-A8 quasispecies is still going through variable dynamic changes. Seemingly, CV-A8 strains are still selecting a type of strain that can adapt mostly to the host environment. It is possible that CV-A8 could become an important pathogen in the HFMD spectrum in the future. Further surveillance of CV-A8 is greatly needed.

## Figures and Tables

**Table 1:** Information on 34 coxsackievirus A8 (CV-A8) complete *VP1* sequences for genotyping.

Genbank No.	Strain ID	Source	Length	Isolated Province (China)	Isolated Country	Isolated Year	Genotype
AF081299	Donovan	Genbank	Full-length	NA	United States	1949	A
KC879489	22468	Genbank	*VP1*	NA	Armenia	2004	B
KC879490	22809	Genbank	*VP1*	NA	Russia	2004	B
KC879494	24267	Genbank	*VP1*	NA	Russia	2005	B
JN203522	N-650	Genbank	*VP1*	NA	Inida	2007	B
JN203523	N-662	Genbank	*VP1*	NA	Inida	2007	B
KC879502	29089	Genbank	*VP1*	NA	Turkmenistan	2007	C
JN203524	N-672	Genbank	*VP1*	NA	Inida	2008	B
JX088583	11186	Genbank	*VP1*	Shandong	China	2011	D
KC867069	JB141230124	Genbank	*VP1*	Guangdong	China	2012	D
KC867070	JB141230127	Genbank	*VP1*	Guangdong	China	2012	D
KC867071	JB141230157	Genbank	*VP1*	Guangdong	China	2012	D
KM609475	SZ93	Genbank	Full-length	Guangdong	China	2012	D
KM609476	SZ124	Genbank	Full-length	Guangdong	China	2012	D
KM609477	SZ127	Genbank	Full-length	Guangdong	China	2012	D
KM609478	SZ141	Genbank	Full-length	Guangdong	China	2012	D
KM609479	SZ157	Genbank	Full-length	Guangdong	China	2012	D
KM609480	SZ39	Genbank	Full-length	Guangdong	China	2013	D
KM609481	SZ40	Genbank	Full-length	Guangdong	China	2013	D
KP289435	P82	Genbank	Full-length	Zhejiang	China	2013	D
MT648779	GS-13-467	This study	Full-length	Guangdong	China	2013	D
KP765687	SZ266	Genbank	Full-length	Guangdong	China	2014	E
MT648780	GS-14-481	This study	Full-length	Gansu	China	2014	E
MT648781	GS-14-943	This study	Full-length	Gansu	China	2014	E
MT648778	CQ-15-Y-120	This study	Full-length	Chongqing	China	2015	E
MT648783	SD-HZ040	This study	Full-length	Shandong	China	2015	D
MT648785	TJ-16-0148	This study	Full-length	Tianjin	China	2016	E
MH111056	C177	Genbank	Full-length	NA	Australia	2017	E
MH111057	C184	Genbank	Full-length	NA	Australia	2017	E
MT648784	SaX-17-77	This study	Full-length	Shaanxi	China	2017	E
MT648787	JX-17-115	This study	Full-length	Jiangxi	China	2017	E
MT648788	JX-17-97	This study	Full-length	Jiangxi	China	2017	E
MT648782	HeN-18-96	This study	Full-length	Henan	China	2018	E
MT648786	TJ-18-38	This study	Full-length	Tianjin	China	2018	C

NA, not applicable.

**Table 2:** Polymorphic amino acid (aa) sites particular to genotype E in *VP1* capsid; the same aa as the genotype A prototype strain is indicated by a dot.

Genotype	Polymorphic Sites								
	25	30	61	* 100	* 103	* 240	* 241	279	282
				BC-Loop		HI-loop			
A	D	N	N	Q	F	A	T	T	T
B	.	.	.	R	.	.	.	.	A
	.	.	.	K	.	.	.	.	A
	.	.	.	.	.	.	.	.	A
	.	.	.	.	.	.	.	.	A
	.	.	.	.	.	.	.	.	A
	.	.	.	.	.	.	.	.	V
C	.	.	.	.	.	.	.	.	.
	.	.	.	.	.	.	.	.	.
D	S	.	.	R	.	.	.	.	.
	S	.	.	.	.	.	.	.	.
	S	.	.	.	.	.	I	.	.
	S	.	.	.	.	.	.	.	.
	G	.	.	.	.	.	.	.	.
	S	.	.	.	.	.	.	.	.
	G	.	.	.	.	.	.	.	.
	S	.	.	.	.	.	I	.	.
	S	.	.	.	.	.	.	.	.
	G	.	.	.	.	.	.	.	.
	G	.	.	.	.	.	.	.	.
	G	.	.	.	.	.	.	.	.
	G	.	.	.	.	.	.	.	.
	G	.	.	.	.	.	.	.	.
E	N	D	S	N	Y	T	V	A	D
	N	D	S	N	Y	T	V	A	D
	N	D	S	N	Y	T	V	A	D
	N	D	S	N	Y	T	V	A	D
	N	D	S	N	Y	T	V	A	D
	S	D	S	N	Y	T	V	.	D
	S	D	S	N	Y	T	V	.	D
	N	D	S	N	Y	T	V	A	N
	N	D	S	N	Y	T	V	A	D
	N	D	S	N	Y	T	V	A	D
	N	D	S	N	Y	T	V	A	D

* Amino acid residues embedded in the potential capsid loops.

## Supplementary Materials

The following are available online at https://www.mdpi.com/1999-4915/12/10/1061/s1, Figure S1: (A) The maximum likelihood phylogenetic tree, (B) neighbor-joining tree, (C) minimum evolution tree and (D) UPGMA tree of CV-A8 strains constructed based on 34 complete VP1 sequences, the CV-A3 prototype strain (AY421761) was used as an outgroup, support was estimated with 1000 bootstrap replicates, Figure S2: The uncollapsed maximum likelihood phylogenetic tree of 171 global CV-A8 partial *VP1* sequences for detailed information, Table S1: Primers for CV-A8 sequencing designed in this study, Table S2: Information on 171 CV-A8 partial VP1 sequences. Chinese isolates were shaded.
