# Supplementary material for: Genetic Diversity Analysis of Coxsackievirus A8 Circulating in China and Worldwide Reveals a Highly Divergent Genotype
Source: Viruses. 2020 Sep 23;12(10):1061. doi: 10.3390/v12101061 (PMC7598191; doi:10.3390/v12101061)
Supplement: Supplementary file 1 [file viruses-12-01061-s001.pdf]

Supplementary materials

Supplementary Table 1. Primers for CV-A8 sequencing designed in this study

Primer name and position	Sequence (5′-3′)	Orientation
CA8-548-A	AACACGGACACCCAAAGTAGTC	Reverse
CA8-446-S	TAGTAGTCCTCCGGCCCCTGAA	Forward
CA8-1369-A	TTTTGCAGTGTTGGGCTTGGG	Reverse
CA8-1150-S	ATGTCTACGCAAATGTGGCAGGA	Forward
CA8-2353-A	GCAGCTCCCAATGCTATTAT	Reverse
CA8-1882-S	GTCAATAATGTGACTGRGCAAG	Forward
CA8-2862-A	ATCACCCTGGGAGTTGTG	Reverse
CA8-2707-S	GGGGTGATCAATGTGCAAGA	Forward
CA8-3752-A	TTGCTCCATCGCCTCCTCAT	Reverse
CA8-3943-S	TGCCAAACTGGAGTGTA	Forward
CA8-4945-A	ACCTTRGATTTCCTGTCTCT	Reverse
CA8-4663-S	TTCATYCCTCCAATGGCTTC	Forward
CA8-5710-A	CCTACYGGCACAAACATTGA	Reverse
CA8-5344-S	CTCAAGAAACCTGTTCTCCGCAC	Forward
CA8-6458-A	GAGTCATTCAAGCTGCTAGCTT	Reverse
CA8-6207-S	TCAGATGAGCATGGAGGAGG	Forward
CA8-7096-A	TCCTTCATTGGCATRGTRGGGTG	Reverse
CA8-6759-S	CAACAAAACCTATTGCGTRCTTGGTG	Forward

Supplementary Table 2. Information on 171 CV-A8 partial *VP1* sequences. Chinese isolates were shaded.

Genbank ID	Isolated Country	Isolated year	Genotype
AF081229	USA	1949	A(prototype)
AB268280	CHN	2000	D
AB167803	JPN	2002	Ungenotyped
AB167804	JPN	2002	Ungenotyped
KC879489	ARM	2004	B
KF412905	IND	2004	B
KC879490	RUS	2004	B
AB727979	CHN	2005	D
KC879494	RUS	2005	B
JQ968946	CHN	2006	D
KF412944	IND	2006	B
JX538081	BAN	2007	Ungenotyped
JN169010	CHN	2007	D
JN203522	IND	2007	B
JN203523	IND	2007	B
KC879502	TKM	2007	C
HQ844645	CHN	2008	D
JN169011	CHN	2008	D
JN203524	IND	2008	B

KJ472834	KEN	2008	Ungenotyped
JQ744287	PHL	2009	Ungenotyped
JQ744288	PHL	2009	Ungenotyped
JX088583	CHN	2011	D
KY885639	CHN	2011	D
MK111159	CYP	2011	E
MK111160	CYP	2011	E
KC893485	NL	2011	E
AB759896	CHN	2012	D
AB759897	CHN	2012	D
KC867069	CHN	2012	D
KC867070	CHN	2012	D
KC867071	CHN	2012	D
KC867072	CHN	2012	D
KC867073	CHN	2012	D
KM609475	CHN	2012	D
KM609476	CHN	2012	D
KM609477	CHN	2012	D
KM609478	CHN	2012	D
KM609479	CHN	2012	D
KY885640	CHN	2012	D
KY885641	CHN	2012	D
MK111161	CYP	2012	E
MK111162	CYP	2012	E
KF383362	THA	2012	E
KF383363	THA	2012	E
KF383364	THA	2012	B
KF383365	THA	2012	B
KF383366	THA	2012	B
KF383367	THA	2012	B
KF661169	THA	2012	B
KF661170	THA	2012	B
KF661171	THA	2012	B
KF661172	THA	2012	B
KF661173	THA	2012	B
KF661174	THA	2012	B
KF661175	THA	2012	B
KF661176	THA	2012	B
KF661177	THA	2012	B
KF661178	THA	2012	B
KF661179	THA	2012	Ungenotyped
KF661180	THA	2012	B
KF661181	THA	2012	B
KF661182	THA	2012	B
KF661183	THA	2012	B
KF661184	THA	2012	B
KF661185	THA	2012	B
KF661186	THA	2012	B
KF661187	THA	2012	B
KF661188	THA	2012	B
KF661189	THA	2012	B
KF661190	THA	2012	B

KF661191	THA	2012	B
KF661192	THA	2012	B
KF661193	THA	2012	B
KF661194	THA	2012	B
KF661196	THA	2012	B
KF661197	THA	2012	B
KF661198	THA	2012	B
KF661199	THA	2012	B
KF661200	THA	2012	B
KF661201	THA	2012	B
KF661202	THA	2012	B
KF661203	THA	2012	B
KF661204	THA	2012	B
KF661205	THA	2012	B
KF661206	THA	2012	B
KF661207	THA	2012	B
GS13-467	CHN	2013	D
KF780574	CHN	2013	D
KF780575	CHN	2013	D
KF780576	CHN	2013	D
KF780577	CHN	2013	D
KM609480	CHN	2013	D
KM609481	CHN	2013	D
KP289435	CHN	2013	D
KY913294	CHN	2013	D
KY913295	CHN	2013	D
AB848735	JPN	2013	E
AB908260	JPN	2013	E
LC124146	JPN	2013	E
KY865769	NL	2013	E
MK307023	CHN	Oct.2013-Sep.2016*	E
MK307024	CHN	Oct.2013-Sep.2016	E
MK307025	CHN	Oct.2013-Sep.2016	E
MK307026	CHN	Oct.2013-Sep.2016	E
MK307027	CHN	Oct.2013-Sep.2016	E
MK307028	CHN	Oct.2013-Sep.2016	E
MK307029	CHN	Oct.2013-Sep.2016	E
MK307030	CHN	Oct.2013-Sep.2016	E
MK307031	CHN	Oct.2013-Sep.2016	E
MK307032	CHN	Oct.2013-Sep.2016	E
MK307033	CHN	Oct.2013-Sep.2016	E
MK307034	CHN	Oct.2013-Sep.2016	E
MK307035	CHN	Oct.2013-Sep.2016	E
MK307036	CHN	Oct.2013-Sep.2016	E
MK307037	CHN	Oct.2013-Sep.2016	E
MK307038	CHN	Oct.2013-Sep.2016	E
MK307039	CHN	Oct.2013-Sep.2016	E
MK307040	CHN	Oct.2013-Sep.2016	E
MK307041	CHN	Oct.2013-Sep.2016	E
MK307042	CHN	Oct.2013-Sep.2016	E
MK307043	CHN	Oct.2013-Sep.2016	E
MK307044	CHN	Oct.2013-Sep.2016	E

MK307045	CHN	Oct.2013-Sep.2016	E
MK307046	CHN	Oct.2013-Sep.2016	E
MK307047	CHN	Oct.2013-Sep.2016	E
MK307048	CHN	Oct.2013-Sep.2016	E
MK307049	CHN	Oct.2013-Sep.2016	E
MK307050	CHN	Oct.2013-Sep.2016	E
MK307051	CHN	Oct.2013-Sep.2016	E
MK307052	CHN	Oct.2013-Sep.2016	E
MK307053	CHN	Oct.2013-Sep.2016	E
MK307054	CHN	Oct.2013-Sep.2016	E
MK307055	CHN	Oct.2013-Sep.2016	E
MK307056	CHN	Oct.2013-Sep.2016	E
MK307057	CHN	Oct.2013-Sep.2016	E
MK307058	CHN	Oct.2013-Sep.2016	E
MK307059	CHN	Oct.2013-Sep.2016	E
MK307060	CHN	Oct.2013-Sep.2016	E
GS14-481	CHN	2014	E
GS14-943	CHN	2014	E
KP765687	CHN	2014	E
KY885642	CHN	2014	D
KY866545	NL	2014	E
MK086333	FRA	2014-2015**	Ungenotyped
MK086334	FRA	2014-2015	Ungenotyped
MK086335	FRA	2014-2015	Ungenotyped
MK086336	FRA	2014-2015	Ungenotyped
CQ15-Y-120	CHN	2015	E
KY885643	CHN	2015	E
KY913296	CHN	2015	E
KY913297	CHN	2015	E
LC120907	CHN	2015	E
SDHZ040	CHN	2015	D
MK086258	FRA	2015	E
MK561377	FRA	2015	E
KY861076	SA	2015	E
KY861077	SA	2015	E
KY861078	SA	2015	E
TJ16-0148	CHN	2016	C
MH111056	AUS	2017	E
MH111057	AUS	2017	E
SAX17-77	CHN	2017	E
MG844064	THA	2017	E
MG844065	THA	2017	E
MG844066	THA	2017	E
MG844067	THA	2017	E
MG844068	THA	2017	E
HEN18-96	CHN	2018	E
MN058705	IR	2018	Ungenotyped
MK836137	USA	2018	Ungenotyped

*no specific isolated year information for these Chinese strains

**no specific isolated year information for these French strains

Supplementary Figures and legends

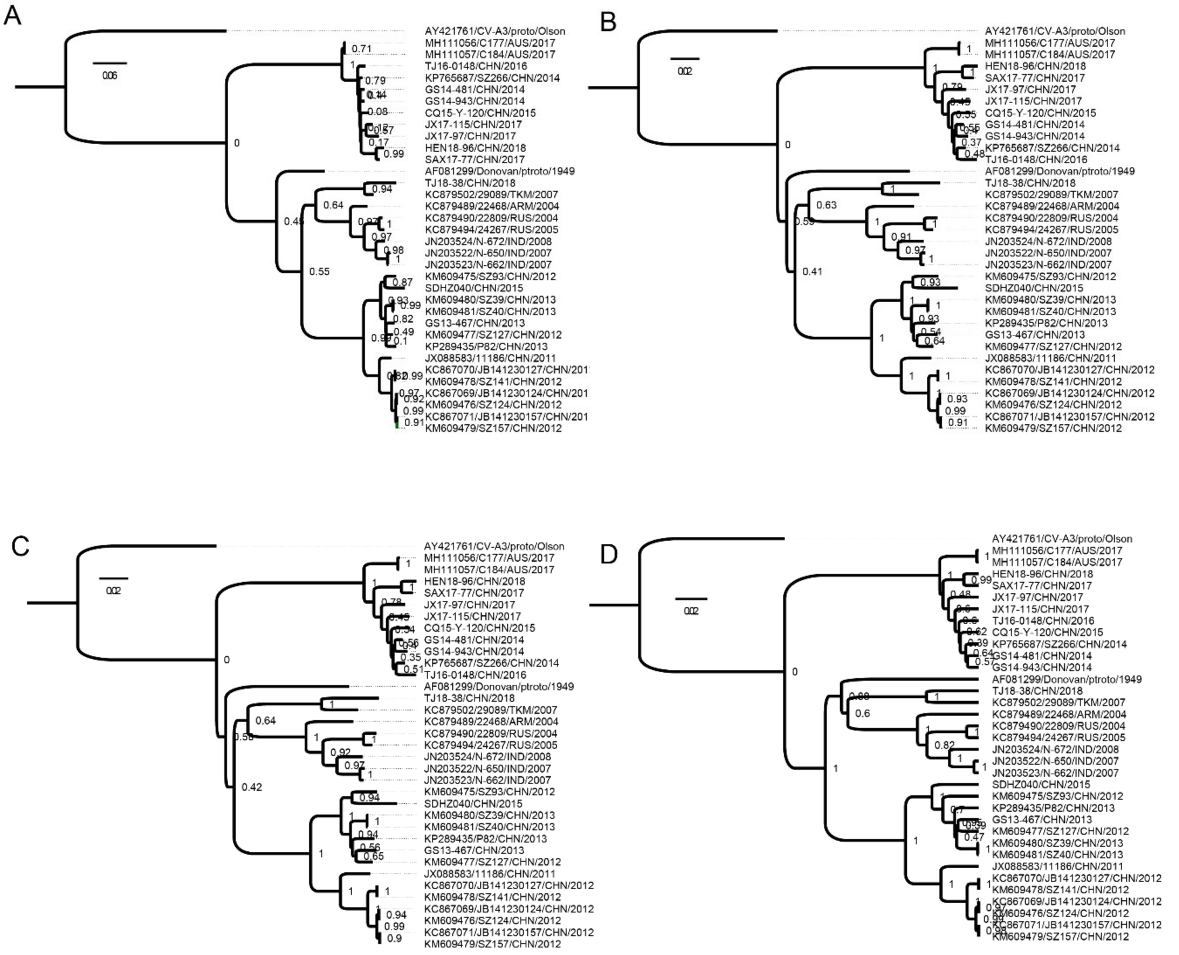

Supplementary Figure 1. (A) The maximum likelihood phylogenetic tree, (B) neighbor-joining tree, (C) minimum evolution tree and (D) UPGMA tree of CV-A8 strains constructed based on 34 complete *VP1* sequences, the CV-A3 prototype strain (AY421761) was used as an outgroup, support was estimated with 1,000 bootstrap replicates.

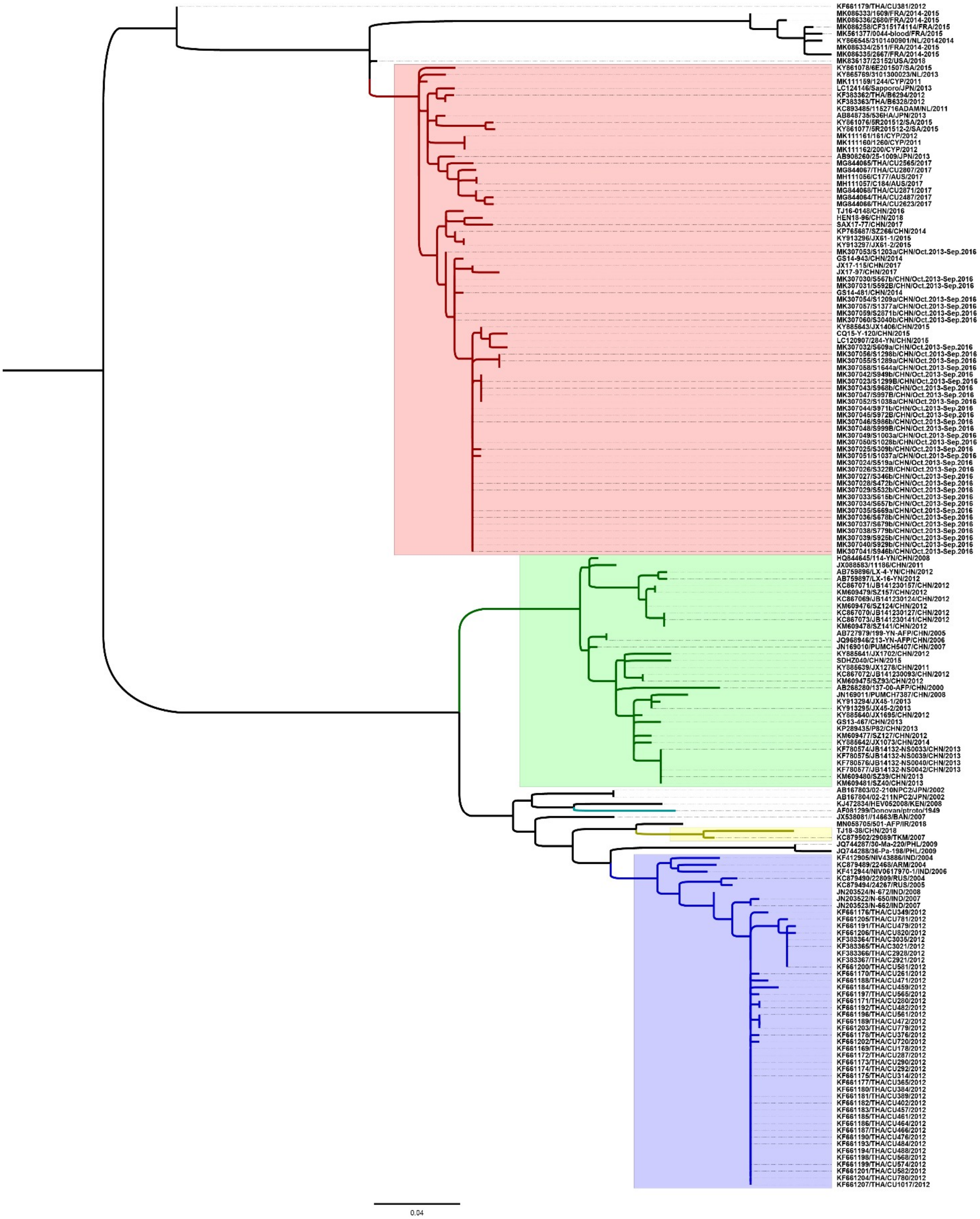

Supplementary Figure 2. The uncollapsed maximum likelihood phylogenetic tree of 171 global CV-A8 partial *VP1* sequences for detailed information.
